# Biocontrol of Crown Gall Disease of Cherry Trees by *Bacillus velezensis*

**DOI:** 10.3390/plants14030475

**Published:** 2025-02-05

**Authors:** Yongfeng Li, Zhaoliang Gao, Weiliang Kong, Yueting Xiao, Mark Owusu Adjei, Ben Fan

**Affiliations:** 1 Co-Innovation Center for Sustainable Forestry in Southern China, College of Forestry and Grassland, Nanjing Forestry University, Nanjing 210037, China; ffzds@njfu.edu.cn (Y.L.); k3170100077@njfu.edu.cn (W.K.); xiaoyueting@njfu.edu.cn (Y.X.); kofiadjeinelson@gmail.com (M.O.A.); 2 Forest & Fruit Tree Research Institute, Shanghai Academy of Agricultural Sciences, Shanghai 201403, China; gaozhaoliang@126.com

**Keywords:** *Agrobacterium tumefaciens*, *Bacillus velezensis*, biological control, ISR, Japanese cherry

## Abstract

Crown gall disease (CGD), caused by *Agrobacterium tumefaciens*, is a common plant disease that leads to significant economic losses. Biological control is a sustainable and scalable method for managing CGD. In this study, we isolated three *Bacillus* strains from the rhizosphere soil of healthy cherry trees and investigated their biocontrol activities and the underlying mechanisms against CGD of cherry trees. The results demonstrate that the three *Bacillus* strains can effectively inhibit the growth of the pathogenic *A. tumefaciens* strain XYT58 in vitro under different culture conditions. The pot experiments showed that the three strains could prevent CGD in cherry seedlings. Using PCR amplification, we identified the genes responsible for the synthesis of difficidin, macrolactin, and bacilysin in the three strains. In addition, inoculation with strains WY66 and WY519 significantly enhanced the expression of JA, ET, and SA pathway-related genes in cherry plants. The presence of antibiotic synthesis-related genes in the *Bacillus* strains and the trigger of plant ISR may explain their ability to control CGD in cherry trees. The findings of this study provide a theoretical basis for the application and development of plant growth-promoting rhizobacteria *Bacillus* strains in the control of CGD.

## 1. Introduction

CGD is a plant bacterial disease caused by *A. tumefaciens*, which infects host plants by transferring its T-DNA into plant cells [1,2,3]. This results in a series of symptoms that disrupt the normal growth of the infected plant [4,5]. The *Agrobacterium* pathogen can survive on plant surfaces or in the soil, remaining viable for up to a year, and be disseminated by underground pests, farming tools, and nursery stock. Many economically important plants, including stone fruits, *Rosaceae* family members, and certain leafy vegetables, are susceptible to *A. tumefaciens* infection [6,7,8], leading to significant economic losses. In the early 21st century, CGD was detected in fruit-growing regions across several countries in North Africa and Western Asia [6]. A study conducted by Polish researchers in 2015 on 80 fruit tree nurseries found that half of these nurseries were infected with CGD [9].

Japanese cherry, recognized as the national flower of Japan, holds significant ornamental value. As a typical plant of the *Rosaceae* family, Japanese cherry trees are highly susceptible to infection by *A. tumefaciens*. In recent years, as the cultivation area of cherry blossoms in China has rapidly expanded and the trees have been transported as ornamental saplings across the country, outbreaks of CGD have been reported in many cherry blossom nurseries [10,11]. This disease has become a major obstacle to the development of the cherry blossom industry in China.

For many years, plant pathologists have dedicated themselves to finding safe and effective methods to control CGD. In the past, chemical pesticides were widely used [12]; however, their negative impact on the environment and ecology is a serious concern [8]. By contrast, biological control is a cost-effective practice, which can provide broad-spectrum protection to different host plants [13,14], making it a preferred method for controlling CGD in plants today [15]. Plant growth-promoting rhizobacteria (PGPR) play a crucial role in biocontrol. PGPR can colonize the roots of plants, competing with harmful microorganisms and soil-borne pathogens [16,17,18]. This process often involves various antibiotics, which protect the host plant from pathogen infection [19,20]. Many strains in the genus *Bacillus* are typical PGPR with a broad spectrum of antimicrobial activity. Due to their ability to produce endospores that are highly tolerant to environmental conditions, *Bacillus* strains have a significant advantage in the preparation and application of products [21]. As a result, many *Bacillus* strains have been successfully developed as commercial biocontrol agents [22,23,24,25]. Many PGPR also inhibit soil-borne diseases through triggering induced systemic resistance (ISR) in plants [26,27]. In this process, PGPR provide preemptive protection for plants when exposed to pathogens [28]. The jasmonic acid (JA) and ethylene (ET) pathways play important roles in ISR, which, meanwhile, has a close cross-talk with the salicylic acid (SA) pathways involved in the systemic acquired resistance (SAR) [16,29,30].

PGPR including *Bacillus* strains have also been used in the biocontrol of CGD. For example, researchers isolated PGPR strains from the rhizosphere of roses, which reduced gall size by 26% [31], and in another study, twelve bacterial strains isolated from the rhizosphere were tested in combination with commonly used antagonistic agents K84 and K1206 against crown gall in stone fruit nurseries [32], and several strains significantly reduced the incidence of the disease. In addition, it is found that the strain *B. albus* JK-XZ3 exhibits a strong antagonistic effect against crown gall in cherry trees [33], while *B. amyloliquefaciens* JK10 can effectively control crown gall in blueberries [34,35].

Although there are studies reporting that isolated PGPR strains are effective in inhibiting crown gall [36], only few focused on the mechanisms by which PGPR strains protect host plants from CGD [37]. We assume that these PGPR strains could also be applied to the control of CGD of cherry trees. This study aims to isolate the strains and investigate their potential effects against the crown gall of Japanese cherry trees and the underlying mechanisms.

## 2. Materials and Methods

### 2.1. Isolates and Strains

To isolate the bacterial strain used for these studies, we first obtained root tumors or galls from Japanese cherry trees located at Ximi Green Plantation in Muyang, Jiangsu Province, China. The surface of the root tumors was sterilized by immersing them in 70% sodium hypochlorite (NaOCl) solution for 1 min and washed twice in 75% ethanol solution and in sterile water, respectively. The tumors were ground into powder after drying and suspended in 100 mL sterile water. The resulting suspension was serially diluted and spread on LB solid medium for bacterial colony growth at 30 °C for 48 h.

### 2.2. Phylogenetic Test

Genomic DNA was isolated from the obtained bacteria using the CTAB method with minor modifications [35]. For the genetic marker to identify a bacterium, the 16S rDNA region was amplified using the primers 27F (5′-AGAGTTTGATCCTGGCTCAG) and 1492R (5′-AAGGAGGTGATCCAGCCGCA). The resulting PCR products were submitted to Sangon Biotech (Shanghai) Co., Ltd. (Shanghai, China) for sequencing. Sequence alignment and a phylogenetic tree were constructed using MEGA 10.0 software.

### 2.3. Antagonism Assay Between Biocontrol Bacteria and Pathogens

The antagonism assay between biocontrol bacteria and pathogens was performed. For the bacterial antagonism assay, the isolated bacterial strains were inoculated in 10 mL liquid LB medium, shaken until the OD_600_ = 1. Then, 0.1% of the culture was inoculated into fresh LB medium and incubated overnight at 30 °C at 200 rpm before 2 μL of overnight bacterial culture was spotted at the center of the pathogen plates using a pipette and allowed to dry and incubated at 30 °C for 48 h.

For the fermentation filtrate antagonism assay, a fresh single colony of the strain was picked and inoculated in 10 mL LB liquid medium and shaken at 37 °C and 200 rpm until an OD_600_ = 1. 0.1% of the inoculum was transferred to 50 mL of Landy liquid medium, incubated at 37 °C and 200 rpm for 24 h. The fermentation broth was collected, centrifuged at 13,000 rpm for 20 min, and the supernatant was filtered using a 0.22 μm sterile filter to obtain sterile fermentation filtrate. The 5 mm hole was punched at the center of the pathogen plate using a sterile puncher, and 50 μL of sterile fermentation filtrate was added to the hole. After drying under the laminar flow hood, the plates were incubated at 30 °C for 48 h. Both assays were photographed, and the size of the inhibition zones (mm) was measured after incubation. Each experiment was triplicated. The inhibition rate (%) was calculated as follows: Inhibition rate (%) = inhibition zone diameter/plate area diameter × 100%.

### 2.4. Biological Assay

For the cherry seedling experiments, bacterial cells of WY14, WY66, and WY519 were harvested by centrifugation and subsequently re-suspended in PBS buffer. The suspensions were adjusted to three final concentrations: 10^7^ CFU/mL, 10^8^ CFU/mL, and 10^9^ CFU/mL, while the XYT58 culture was standardized to a concentration of 10^8^ CFU/mL. The seedlings were divided into three groups, each containing ten plants. The residual soil on the roots was carefully washed off, and three wounds, 1 cm in length and 2 mm in depth, were randomly made on both the main and lateral roots using a sterile scalpel. Equal volumes (10 mL) of the prepared three-strain suspension and the XYT58 culture were applied to the wounds by immersing the roots. The inoculated root areas were wrapped to retain moisture, and the seedlings were transplanted into nursery pots. After 60 days, the incidence of disease in the seedlings was recorded. The diameters of the top 10 tumors formed on each plant were measured, and the mortality rates for each group were calculated. A control group of 30 seedlings, inoculated with XYT58 but not treated with *Bacillus*, was used for comparison. The biocontrol efficacy (%) of these three strains against *Agrobacterium* was calculated using the following formula: Biocontrol efficacy (%) = (Disease index of CK − Disease index of treatment)/Disease index of CK × 100%.

### 2.5. Amplification of the Surfactin Synthesis Gene Sequence

The surfactin synthesis gene sequence was amplified using primers designed for the antibiotic synthesis gene (Table 1). Genomic DNA extracted from the isolated strain was used as a template for the PCR reaction. The reaction mixture (25 µL total volume) consisted of 1 µL of each primer (forward and reverse), 2 µL of template DNA, 12.5 µL of T-mix (Ampliqon, Denmark), and 8.5 µL of sterile water. The PCR conditions were as follows: initial denaturation at 94 °C for 5 min, followed by 35 cycles of denaturation at 95 °C for 1 min, annealing at 55 °C for 1.5 min, and extension at 72 °C for 1.5 min, with a final extension at 72 °C for 10 min. PCR products were analyzed and observed by gel electrophoresis using 1.5% agarose gel in TBE buffer.

### 2.6. RT-PCR

Gene expression levels were quantified using the 2-∆∆Ct method. Each gene was analyzed with three technical replicates and five biological replicates. The primer sequences used for RT-PCR are listed in Table 2.

### 2.7. Enzyme Activity Assay

Fresh seedling (Name of seedling) leaves (0.5 g) were placed in a mortar, and 2–3 mL of pre-cooled PBS buffer (pH 7.0) at 4 °C along with a small amount of quartz sand were added. The mixture was ground into a homogenate, and all was collected into a 25 mL volumetric flask. The final solution was adjusted to 25 mL, mixed thoroughly, and left to stand in a refrigerator at 5 °C for 10 min. Afterward, the solution was centrifuged at 4000 rpm for 15 min. The resulting supernatant, which constituted the crude enzyme extract, was stored at 5 °C for subsequent analysis. The activities of superoxide dismutase (SOD), peroxidase (POD), and catalase (CAT) were determined using previously established methods.

### 2.8. Statistical Analysis

The diameters of galls (Table 2) and the inhibition zones were compared using one-way ANOVA analysis. The statistical analyses for the differentially expressed genes and the changes in the antioxidant enzyme activity were performed using Student’s *t*-test.

## 3. Results

### 3.1. Three Bacillus Strains Inhibiting the Growth of Agrobacterium Were Isolated

**Figure 1:**
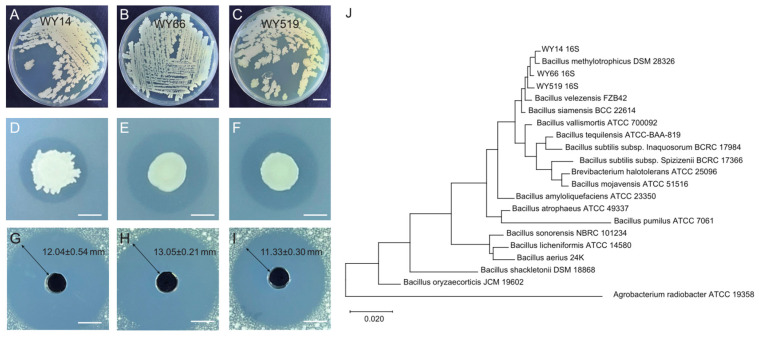
Inhibitory effects of three *Bacillus* strains on *A. tumefaciens* XYT58 and the phylogenetic tree based on their 16s rDNA sequences. Morphology of the streaks (**A**–**C**) and colonies (**D**–**F**) of the three strains (WY14, WY66, and WY519) on LB plates; (**G**–**I**) Inhibitory zones of *A. tumefaciens* XYT58 by the sterile culture filtrates of the three *Bacillus* strains. (**A**,**D**,**G**): WY14; (**B**,**E**,**H**): WY66; (**C**,**F**,**I**): WY519. (**J**) Phylogenetic tree constructed based on the 16s rDNA sequences of the three strains.

Using *A. tumefaciens* XYT58, previously isolated from the crown gall of a cherry tree, as the pathogen, we isolated three strains (WY14, WY66, and WY519) with strong antagonistic effects. Strain WY14 exhibited irregular colony morphology with wavy edges, while strains WY66 and WY519 formed circular colonies with regular edges (Figure 1A–F). All three strains tested positive for Gram staining.

To more precisely compare the antagonistic activity of the three strains, we prepared sterile culture filtrates of the strains and evaluated their effect on the growth of XYT58. WY66 exhibited the strongest prevention effect, with an inhibition zone diameter of 13.5 ± 0.21 mm. WY519 showed moderate inhibition with a zone diameter of 12.33 ± 0.30 mm, while WY14 had the weakest effect with an inhibition zone diameter of 12.04 ± 0.54 mm (Figure 1G–I). We performed phylogenetic analysis of the strains WY14, WY66, and WY519 using their 16S rDNA sequence. The results indicate that all the three strains belong to the *Bacillus* genus, and the most homologous strain was *B. velezensis* FZB42 (Figure 1J).

### 3.2. Biocontrol Effect of Antagonistic Bacteria on CGD in Potted Cherry Seedlings

**Figure 2:**
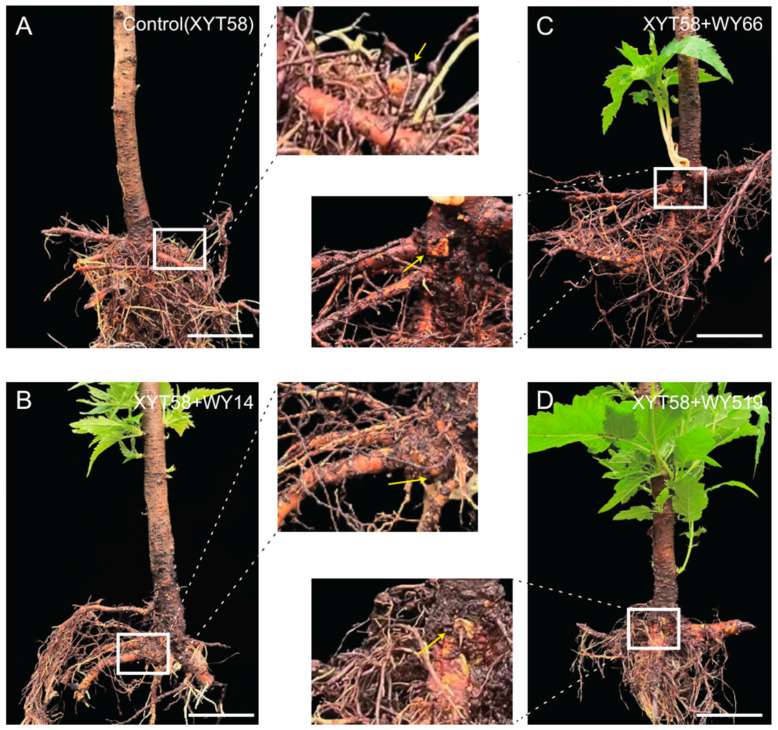
Effect of inoculation with the *Bacillus* strains on the growth of cherry seedlings. The representative seedlings in the non-inoculated control (**A**) and in the treatment group (**B**–**D**) inoculated with WY14 (**B**), inoculated with WY66 (**C**), and inoculated with WY519 (**D**).

To evaluate their biocontrol efficacy on plants, we conducted experiments using cherry seedlings. The results show that the control group (without the *Bacillus* strains) exhibited typical disease symptoms, with obvious tumor-like galls on the roots (Figure 2A). In contrast, the seedlings treated with the three *Bacillus* strains displayed milder symptoms of CGD, with less noticeable tumor formation (Figure 2B–D).

The biocontrol efficacy may vary depending on the inoculation number of the biocontrol strains. To evaluate the possible different effect due to inoculum, we pre-inoculated cherry seedlings with different amounts of the *Bacillus* cells some hour before applying *A. tumefaciens* XYT58. The incidence of CGD of the cherry trees was compared 60 days after the inoculation. Compared with the control group without *Bacillus* inoculation, the application of the three *Bacillus* strains significantly reduced both the incidence and severity of CGD in potted cherry seedlings (Table 1). Specifically, when the bacterial concentration reached 10^7^ CFU/mL, the relative control efficacy exceeded 60%; when the concentration reached 10^8^ CFU/mL or higher, the control efficacy reached 100% (Table 3). According to the mortality rate of cherry seedlings, it seems that 10^7^ CFU/mL is a threshold concentration of the *Bacillus* inoculation needed for effective bio-control. However, what the optimal concentration for the application is considering both the mortality rate of cherry seedlings and the diameter of the root nodules still requires further experimental verification. The tumor diameters in the treated groups were all smaller than those in the diseased control group (Table 3). The application of strains WY66 and WY519 reduced the average tumor diameter by more than 20 mm compared to the control group. Furthermore, as the inoculation concentration increased, the tumor size decreased (Table 3). These results demonstrate that the pre-inoculation with strains WY14, WY66, and WY519 can effectively control the occurrence of CGD in cherry seedlings.

### 3.3. The Bacillus Strains Produce Antibiotics That May Inhibit A. tumefaciens

**Figure 3:**
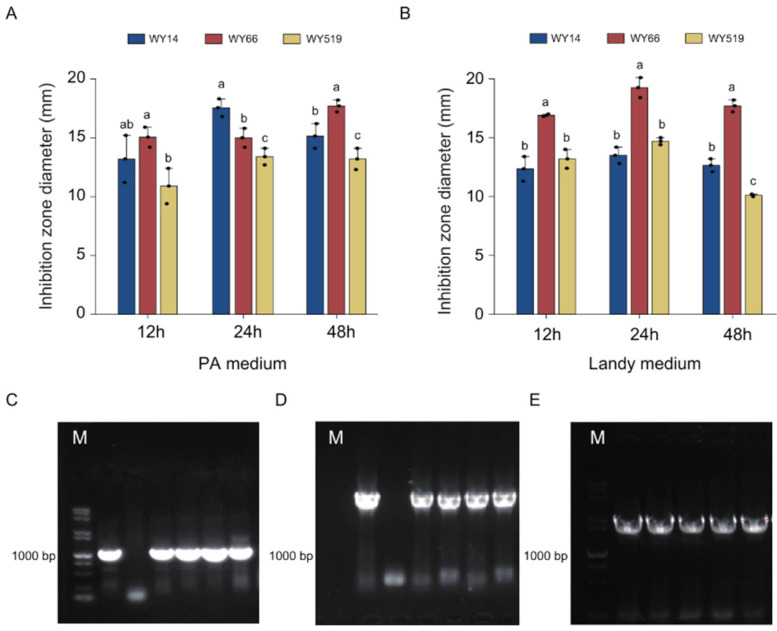
Different antibiotic production of the three *Bacillus* strains may be responsible for the inhibition of *A. tumefaciens* XYT58. (**A**,**B**) The diameter of inhibition zones formed by the supernatant filtrates of the three *Bacillus* strains from different culture times in different media, namely PA medium (**A**) and Landy medium (**B**) against XYT58. (**C**–**E**) Gel electrophoresis of the genes related to antibiotic production amplified from the three *Bacillus* strains. The PCR product of (**C**) the *dfnM* gene, (**D**) the *bacC* gene, and (**E**) the *mlnI* gene amplified from the *B. velezensis* FZB42 genome (the positive control) (lane 1), the no DNA template control (the negative control) (lane 2), and the genome of WY14 (lane 3), WY66 (lane 4), and WY519 (lane 5). Lane M: DNA size marker. One-way ANOVA was used, df = 2. Different letters in (**A**,**B**) indicate significant difference.

We tested the effect of the three *Bacillus* strains on the inhibition of XYT58 under different culture media and incubation periods. The results show that all the cultures of the three strains in both PA medium and Landy medium, two media which are often used in the biocontrol investigations of *B. velezensis* FZB42, exhibited inhibitory activity against XYT58. In the PA medium, the supernatant filtrate of strain WY14 cultured for 24 h displayed the highest activity, with the average diameter of the inhibition zones of 17.84 mm (Figure 3A), while in Landy medium, the supernatant filtrate of strain WY66 cultured for 24 h exhibited the strongest antagonistic activity, with the average diameter of the inhibition zone of 18.38 mm (Figure 3B) These results suggest that the three *Bacillus* strains may produce different antibiotics against XYT58. Given the complexity of the soil environment, a single type of culture medium may not achieve the maximum biocontrol effect of the strains. In future studies, we will further investigate the inhibitory effects of the biocontrol strains WY14 and WY66 when applied in combination.

Using PCR, we amplified some genes for the synthesis of polyketide compounds difficidin and macrolactin, as well as the dipeptide bacilysin, which have been shown to be effective in controlling CGD [38,39,40,41]. We successfully obtained a 1300 bp fragment of the *dfnM* gene for difficidin synthesis (Figure 3D), a 1500 bp fragment of the *bacC* gene involved in bacilysin synthesis (Figure 3E), and a 1300 bp fragment of the *mlnI* gene associated with macrolactin synthesis (Figure 3E). These results indicate that strains WY14, WY66, and WY519 can produce polyketide antibiotics difficidin and macrolactin, as well as the dipeptide antibiotic bacilysin.

### 3.4. Inoculation with the Bacillus Strains Triggers Plant ISR

To determine whether the ISR mechanism contributes to the biocontrol efficacy of the three *Bacillus* strains against CGD, we performed qPCR analysis of defense gene expression in rose seedlings.

**Figure 4:**
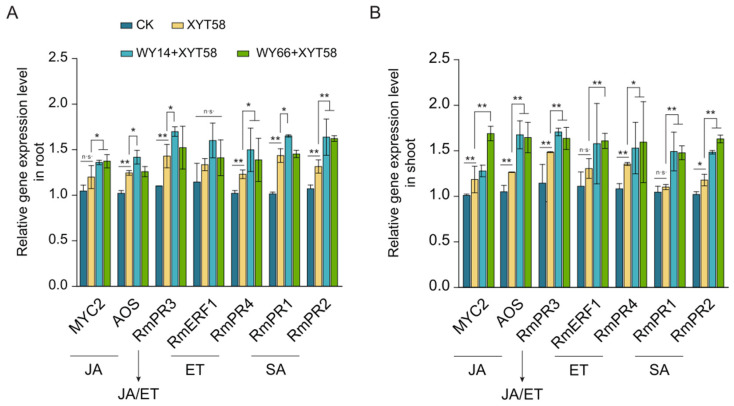
Effect of the inoculation of *B. velezensis* WY14 and WY66 on the expression of the genes related to ISR in the roots (**A**) and the shoot (**B**) part of rose seedlings. Student’s *t*-test was used for the statistics. *: *p* < 0.05; **: *p* < 0.01; n.s.: not significant.

The results show that, in the seedling roots (Figure 4A) in the treatment group, compared to the blank control group (without pathogen or three *Bacillus* strains), the expression of key genes involved in the jasmonic acid (JA) signaling pathway was upregulated by approximately 1.3-fold in the group inoculated with the pathogen XYT58, while genes related to the salicylic acid (SA) and ethylene (ET) pathways were upregulated by around 1.4-fold. This suggests that the pathogen invasion triggered the plant’s systemic acquired resistance (SAR). In the groups pre-inoculated with WY14 and WY66, the expression of these resistance genes was significantly increased. A similar pattern was observed in the aerial parts of the plants (Figure 4B). These results indicate that the *B. velezensis* WY14 and WY66 indeed triggered the ISR mechanism in plants.

### 3.5. No Significant Change Is Detected in the Oxidase Activities of Plants When Inoculated by the Bacillus Strains

**Figure 5:**
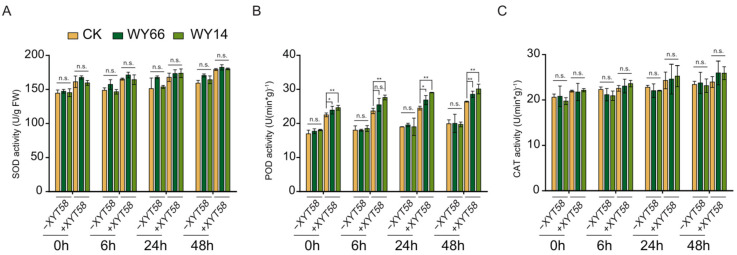
Effect of the inoculation with *B. velezensis* WY14 and WY66 on the activities of the antioxidant enzymes in rose seedlings. (**A**) SOD activity. (**B**) POD activity. (**C**) CAT activity. Student’s *t*-test was used for the statistics. *: *p* < 0.05; **: *p* < 0.01; n.s.: not significant.

We measured the activities of some antioxidant enzymes involved in the defense of plants against the infection of pathogens. The results show that, in the absence of *A. tumefaciens* XYT58, treatment with WY14 or WY66 did not induce sustained accumulation of POD or CAT activities at 6, 24, or 48 h (Figure 5A–C). However, within 48 h after infection with *A. tumefaciens*, CAT and SOD activities were slightly increased in all groups, but no significant differences were observed. In addition, POD activity was significantly enhanced following the inoculation of XYT58; however, the application of the two *Bacillus* strains alone did not result in any significant difference in the POD activity. These results show that the *Bacillus* strains could not strongly affect activities of the plant resistance-related antioxidant enzymes.

## 4. Discussion

In this study, three *Bacillus* species strains were isolated and identified from the rhizosphere soil of healthy cherry trees. In vitro confrontation assays on plates showed that these strains inhibited the growth of *A. tumefaciens* strain XYT58, a previously isolated pathogen for CGD of cherry trees. Further pot trials under different inoculation amounts confirmed that these *Bacillus* strains could directly suppress the incidence of CGD. Additionally, we found that all the three strains harbor genes responsible for the synthesis of difficidin, macrolactin, and bacilysin, and they were able to enhance the expression of genes related to the JA, ET, JA/ET, and SA signaling pathways in plants, indicating that they triggered ISR against *A. tumefaciens*.

The search for safe and sustainable methods to control CGD has been a research focus for plant pathologists worldwide. With the advancement of biotechnology and the growing concerns on environmental protection, chemical control methods have gradually fallen out of favor. Instead, the development of new crown gall-resistant plant varieties and biological control measures are being increasingly employed. However, breeding resistant varieties is time-consuming and costly, as each species requires separate breeding efforts. In contrast, the use of biocontrol strains offers a rapid and effective solution, applicable across different species, making it a valuable approach to controlling CGD. PGPR are bacteria that live in the soil around plant roots or on the root surface. These bacteria, especially *Bacillus*, can promote plant growth and help plants effectively resist pathogenic attacks, making PGPR a key source of biocontrol strains. *Bacillus* strains MBY2 and 32a have been shown to significantly mitigate crown gall disease in dicotyledonous plants, including tomatoes, highlighting their potential as valuable resources for biological control agents [21,42]. The PGPR model strain FZB42 is known to produce multiple antibiotics, including surfactin, bacilysin, difficidin, and macrolactin [38,43,44]. Among them, surfactin and macrolactin having been reported to inhibit *A. tumefaciens* [40,45]. In our results, all three isolated probiotic strains were identified as belonging to the *Bacillus* genus and were found to possess the biosynthetic genes for difficidin, bacilysin, and macrolactin, which aligns well with our expectations. It is not known how these strains differ in their ability to biosynthesize these antibiotics in nature. Given the complexity of soil conditions, the application of a mixture of the *Bacillus* strains may be an advantage considering their possible different levels of antibiotic production. This needs to be studied in the future.

The suppression of pathogens by PGPR strains involves both direct and indirect mechanisms. The three isolated *Bacillus* strains were able to significantly inhibit the growth of *A. tumefaciens* strain XYT58, and the genes related to the biosynthesis of the antibiotics were amplified, suggesting that these strains inhibit XYT58 growth by the direct mechanism. On the other hand, PGPR can induce systemic resistance in plants, enabling them to actively defend against pathogen invasion. Antibiotics secreted by *Bacillus* species have been known to activate ISR in host plants, which may thereby enhance resistance to *A. tumefaciens*. Activation of plant ISR is associated with the transcriptional upregulation of JA, SA, and ET regulatory genes, which confers resistance even in regions distant from the initial site of infection. In this study, co-inoculation of probiotics and pathogens in cherry saplings resulted in detectable changes in the transcription of genes related to the JA, SA, and ET pathways in both root and distal aerial tissues. This indicates that the colonization of the probiotic *Bacillus* strains also led to the activation of systemic resistance in the plants.

Interestingly, we observed a significant increase in the transcription of the root-specific *PmPR1* gene upon infection with strain XYT58, likely due to the activation of the plant’s SAR mechanism by the pathogen. It is not clear whether this is related to the crosstalk between JA and SA signaling pathways. The ISR mechanism may enhance plant resistance by regulating reactive oxygen species (ROS) burst [46]. However, in our study, no significant changes in the activity of antioxidant enzymes were observed following the co-inoculation of probiotics and pathogens in the host plants. This discrepancy could be attributed to factors such as sampling time, and further investigations are required to clarify this aspect.

Here, we isolated *Bacillus* strains exhibiting strong inhibitory effects against *A. tumefaciens*. Then, we investigated their mechanisms of inhibition against XYT58 and found that they possess genes related to antibiotic synthesis and can activate the host plant’s ISR mechanism. However, many questions remain to be addressed in future studies. For instance, what is the optimal concentration of the *Bacillus* inoculation for their application? Can these PGPR strains cure or alleviate disease symptoms in plants that have already been infected with crown gall? Further studies on these questions could provide a theoretical foundation for the development of *Bacillus* PGPR-based biological control of plant CGD.

## Figures and Tables

**Table 1:** Primers used in PCR.

Antibiotic	Sequence (5′-3′)	Expected Amplified Fragment Length	Positive Control
difficidin	dfnMF1: CGGAGTGAAACCGTGCCGGGATAAAGA;	~1300	*B. velezensis* FZB42
	dfnMR1: GACCATTCAGAGCGGAAAGCTCC		
bacilysin	bacC1: GGAAACCGCCGTTTACGTTC;	~1500	*B. velezensis* FZB42
	bacC2: CCATGAGGCACACGAAGAGA		
macrolactin	mlnIF1: GGAAGAAAAACAGTCGAGGCGATGCTG;	~1300	*B. velezensis* FZB42
	mlnIR1: GAGAAGCTCCGCCGTCACCAGTG		

**Table 2:** Primers used in RT-PCR.

Name	Sequence (5′-3′)	Gene Product
RmACT-F	TACAACTGGTATTGTCCTGGAC	actin
RmACT-R	TCATGTCCCTGACTATTTCTCG	
RmERF1-F	GATCGCAACCGGTTAGAATAAC	ethylene-responsive transcription factor 1
RmERF1-R	CTCGTCTCAACCATCTCTACTC	
RmPR1-F	CAGTGTGAACTTCTTACGGTTC	pathogenesis-related protein 1
RmPR1-R	TTGCTACGAGTTCATACTGTGT	
RmPR2-F	AATCAAAGTTTCCACAGCCATC	b-1,3-glucanase
RmPR2-R	GAAGAGAGCATAATCGAGACGA	
RmPR3-F	TGGGGTTATTGCTTTGTCAATG	basic chitinase
RmPR3-R	GGTTTGTTTGATTGTGGAGTCA	
RmPR4-F	GGAAAACAATGCGTCGTGTTTT	hevein-like protein
RmPR4-R	TCTCACGTTAGTAGCACTTTGT	
RmAOS-F	ACTACCAGAGACTCTACGACTT	allene oxide synthase
RmAOS-R	TGGGAAATAAAAGCTTCATGCC	
RmMYC2-F	TTCAACCAGGAGACGCTTATG	transcription factor MYC2
RmMYC2-R	CGGAAATCAAAGAGTTGAGGTC	

**Table 3:** Biocontrol effect of the *Bacillus* strains (WY14, WY66, and WY519) on crown galls disease caused by *A. tumefaciens* XYT58 on cherry seedlings.

Treatment	Inoculum Size (CFU/mL)	Dead	Survival	Mortality of Group (%)	Mean Diameter of Galls (mm)	Disease Index	Biocontrol Efficiency (%)
CK	-	11	4	73.33	38.4 ± 8.77 ^a^	3.85	-
WY14	10^7^	4	11	26.67	17.03 ± 5.21 ^bc^	2	69.43%
	10^8^	0	15	0.00	20.36 ± 4.90 ^b^	1.33	100%
	10^9^	0	15	0.00	6.93 ± 1.33 ^c^	0.67	100%
WY66	10^7^	2	13	13.33	19.1 ± 6.08 ^b^	1.67	84.73%
	10^8^	0	15	0.00	13.93 ± 5.67 ^bc^	1.33	100%
	10^9^	0	15	0.00	6.7 ± 2.20 ^c^	0.33	100%
WY519	10^7^	2	13	13.33	18.33 ± 7.10 ^bc^	1.67	84.73%
	10^8^	0	15	0.00	28.86 ± 9.14 ^ab^	2.66	100%
	10^9^	0	15	0.00	16.3 ± 5.23 ^bc^	1.33	100%

Note: The different letters indicate significant differences. One-way ANOVA was used, df = 9.

## Data Availability

The original contributions presented in this study are included in the article. Further inquiries can be directed to the corresponding author.

## Abbreviations

ANOVA, Analysis of Variance; CAT, catalase; CFU, colony forming unit; CGD, crown gall disease; ET, ethylene; ISR, induced systemic resistance; JA, jasmonic acid; PCR, polymerase chain reaction; PGPR, plant growth-promoting rhizobacterium; POD, peroxidase; SA, salicylic acid; SOD, superoxide dismutase; VOC, volatile organic compounds.
